# Nuclear DJ-1 Regulates DNA Damage Repair via the Regulation of PARP1 Activity

**DOI:** 10.3390/ijms24108651

**Published:** 2023-05-12

**Authors:** Zhong-Xuan Wang, Yi Liu, Yao-Lin Li, Qiao Wei, Rong-Rong Lin, Ruiqing Kang, Yang Ruan, Zhi-Hao Lin, Nai-Jia Xue, Bao-Rong Zhang, Jia-Li Pu

**Affiliations:** Department of Neurology, Second Affiliated Hospital, School of Medicine, Zhejiang University, Hangzhou 310009, China; 22018220@zju.edu.cn (Z.-X.W.);

**Keywords:** Parkinson’s disease, DNA damage response, DNA double-strand breaks repair, DJ-1, PARP1

## Abstract

DNA damage and defective DNA repair are extensively linked to neurodegeneration in Parkinson’s disease (PD), but the underlying molecular mechanisms remain poorly understood. Here, we determined that the PD-associated protein DJ-1 plays an essential role in modulating DNA double-strand break (DSB) repair. Specifically, DJ-1 is a DNA damage response (DDR) protein that can be recruited to DNA damage sites, where it promotes DSB repair through both homologous recombination and nonhomologous end joining. Mechanistically, DJ-1 interacts directly with PARP1, a nuclear enzyme essential for genomic stability, and stimulates its enzymatic activity during DNA repair. Importantly, cells from PD patients with the DJ-1 mutation also have defective PARP1 activity and impaired repair of DSBs. In summary, our findings uncover a novel function of nuclear DJ-1 in DNA repair and genome stability maintenance, and suggest that defective DNA repair may contribute to the pathogenesis of PD linked to DJ-1 mutations.

## 1. Introduction

Parkinson’s disease (PD), a very common neurodegenerative disease, is characterized by selective loss of dopaminergic (DA) neurons in the substantia nigra and the presence of intraneuronal inclusions named Lewy bodies [1,2]. Although the pathogenesis of PD remains unclear so far, it is clear that its development is related to a complex interaction between environmental and genetic factors [2,3]. The vast majority of patients with PD are sporadic, but there is still a familial aggregation in 10% to 15% of patients [4,5]. Currently, more than 20 causative genes have been reported to contribute to familial PD, including SNCA, LRRK2, DJ-1, PRKN, and PINK1 [4,5]. Studies of genetic defects and disease-associated proteins have provided us with a window to understand the pathogenic mechanism of PD [6].

Recently, increasing evidence suggests that DNA damage and repair deficiency are implicated in age-related neurodegenerative diseases, including PD [7,8,9,10]. Notably, a well-established association between DNA damage and neurodegeneration has long existed [7,11]. Evidence for a causal role of DNA damage in the onset and progression of neurodegenerative diseases comes from patients and animal models with mutations in DNA damage-responsive genes [7,12]. Persistent DNA damage and defective DNA repair have been reported to trigger a cascade of negative outcomes, such as genome instability, epigenetic alterations, compromised mitochondrial function, proteostatic stress, senescence, and, eventually, cell death [13,14]. In PD, evidence of DNA damage has been reported for decades [15]. Compared to healthy controls, there has been a significant upregulation of DNA damage reported, including oxidized DNA damage and double-strand breaks (DSBs), in DA neurons of PD patients [9,15]. Additionally, the ability of DNA repair, including DSB repair as well as nucleotide excision repair (NER), was found to be significantly impaired in the cells of PD patients [15]. On the other hand, DNA damage has been shown to be associated with PD pathology in mouse models of the disease [16,17,18]. More importantly, mice lacking DNA repair proteins can recapitulate some of the disease phenotype of PD [9,18,19,20]. For instance, mice lacking ATM, an essential part of DSB repair, exhibited PD-like motor abnormalities, as well as reduced expression of tyrosine hydroxylase and dopamine transporters [19,21]. Moreover, ERCC1 mutant mice have been shown to display age-related loss of DA neurons, elevated levels of phospho-α-synuclein, increased astrocyte activation, and mitochondrial dysfunction, and to be more susceptible to damage induced by the neurotoxin 1-methyl-4-phenyl-1,2,3,6-tetrahydropyridine (MPTP) [18]. ERCC1 is a critical player in NER, and without it, the balance between DNA damage and repair would be lost, leading to DNA damage accumulation [18,22]. Thus, the above evidence suggests that unrepaired DNA damage may be a key mechanism for neurodegeneration in PD [9]. However, the underlying mechanisms responsible for the defective DNA repair in PD remain enigmatic.

Loss-of-function mutations in DJ-1 are one of the genetic causes of autosomal recessively inherited PD [6,23]. Furthermore, defects in DJ-1 have also been implicated in sporadic PD and other age-related neurodegenerative diseases [23]. DJ-1 is a 189-amino acid protein that exists mainly as a homodimer in the cytoplasm, mitochondria, and nucleus [23]. Extensive studies have deciphered the roles and mechanisms of cytoplasmic and mitochondrial distributions of DJ-1 [23,24]. It has been shown that DJ-1 in the cytoplasm acts mainly as an oxidative response sensor against oxidative stress [25]. DJ-1 is also reported to be involved in other biological processes, such as signal transduction, protein synthesis, endoplasmic reticulum stress, and chaperone activity [23,25]. Mitochondrial localization of DJ-1 plays an important role in mitochondrial homeostasis, including mitochondrial bioenergetics, dynamics, and motility [23]. Though DJ-1 in the nucleus takes up a considerable abundance of its whole distribution, to date, the exact role of DJ-1 in the nucleus remains shrouded in great mystery [23,26]. Interestingly, recent studies have shown that DJ-1 downregulation increases susceptibility to ionizing radiation, while DJ-1 overexpression protects cells from death induced by etoposide, both of which can induce DSBs [27,28]. In addition, DJ-1 knockout (KO) mice also exhibit elevated DSBs [29], implying a potential role of DJ-1 in maintaining genome stability and repairing DNA DSBs. In this study, we sought to ascertain whether DJ-1 deficiency affects DNA repair and to explore the potential molecular mechanisms.

Here, we found that in mammalian cells subjected to genotoxic insults DJ-1 responds to DNA damage and stimulates DSB repair via direct interaction with PARP1. Moreover, we also demonstrated that the mutation of DJ-1 in PD patients affects PARP1 activity and the subsequent DNA repair. Our findings revealed a previously unrecognized function and mechanism whereby the nuclear distribution of DJ-1 is related to DNA damage and repair.

## 2. Results

### 2.1. DJ-1 Responds to DNA Damage and Is Recruited to Sites of DSBs

**Figure 1:**
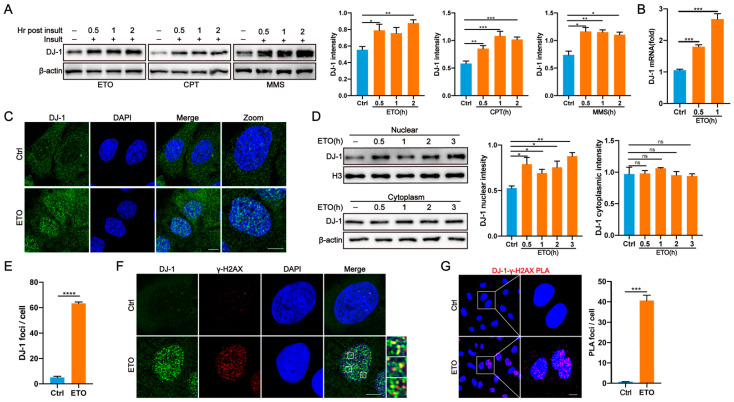
DJ-1 responds to DNA damage and localizes to DSB sites. (**A**) Representative western blot and quantification of DJ-1 in U2OS cells treated with ETO (10 µM), CPT (10 µM), and MMS (0.01%) at the indicated timepoints. n = 3. *, *p* < 0.05; **, *p* < 0.01; ***, *p* < 0.001 (two-tailed *t*-test). (**B**) Transcriptional analysis of DJ-1 in U2OS cells treated with ETO (10 µM) at the indicated timepoints by qPCR. GAPDH was used to normalize expression. Data are presented as the mean ± SEM, n = 3. ***, *p* < 0.001 (two-tailed *t*-test). (**C**) Representative immunofluorescence images of DJ-1 in U2OS cells exposed to ETO (10 µM) for 1 h. Scale bar, 10 µm. n = 3. (**D**) Representative western blot and quantification of endogenous DJ-1 in the cytoplasmic and nuclear fractions of U2OS cells treated with ETO (10 µM) at the indicated times. n = 3. *, *p* < 0.05; **, *p* < 0.01; ns, nonsignificant (two-tailed *t*-test). (**E**) Representative quantification of DJ-1 foci as in C. Data are presented as the mean ± SEM, n > 50 cells. ****, *p* < 0.0001 (two-tailed *t*-test). (**F**) Representative immunostaining of γ-H2AX and DJ-1 following ETO (10 µM, 30 min) treatment in U2OS cells. Scale bar, 10 μm. n = 3. (**G**) Representative PLA signals between γ-H2AX and DJ-1 in U2OS cells following ETO (10 µM, 30 min) treatment. Quantification of average PLA signals is shown at right. Scale bar, 10 μm. Data are presented as the mean ± SEM, n > 50 cells. ***, *p* < 0.001 (two-tailed *t*-test).

To detect and repair damaged DNA, cells have evolved a DNA damage response (DDR) system [30]. DJ-1 is stress response protein [23]. To characterize the effect of DJ-1 in repairing DSBs, we first investigated whether DJ-1 responds to DNA damage. To achieve this, we treated cells with etoposide (ETO), a topoisomerase II inhibitor, which is used to induce DNA DSBs [31,32], and we analyzed DJ-1 protein levels at a series of timepoints. For this experiment, primary human skin fibroblasts, U2OS and M17 cell lines, were treated to ensure that the observed response was not due to specificity within a single cell type. In all cell types, we observed a dramatic induction of DJ-1 protein levels after ETO treatment (Figure 1A and Supplementary Figure S1A,B). Furthermore, similar results were obtained when cells were exposed to camptothecin (CPT) and methyl methanesulfonate (MMS), which can also induce DSBs [31,33] (Figure 1A and Supplementary Figure S1A,B). Then qPCR revealed a significant increased DJ-1 mRNA level induced by ETO treatment (Figure 1B), indicating that DNA damage-induced expression of DJ-1 occurred at the transcriptional level. 

After DNA damage, many DNA repair proteins change their subcellular localization and form nuclear foci at DNA damage sites [34]. In unperturbed U2OS cells, immunofluorescence studies showed that DJ-1 was mainly localized in the cytoplasm, and also abundantly expressed in the nucleus with a diffuse distribution (Figure 1C), which is consistent with previous reports [26]. However, compared to the control condition, exposure to ETO resulted in an obvious nuclear distribution of DJ-1, as confirmed by immunofluorescence and subcellular fractionation analysis (Figure 1C,D). Importantly, numerous DJ-1 nuclear foci were observed in the nucleus after ETO treatment, suggesting that DJ-1 may have a direct effect on DNA damage repair (Figure 1E).

Next, we investigated whether DJ-1 was recruited to DNA damage sites. γH2AX foci formation is a rapid initial event in the cellular response to DSBs, which works as an essential scaffold for other recruited repair proteins at the site of DSBs [34,35]. In cells treated with ETO, immunofluorescence revealed an obvious colocalization between DJ-1 and γH2AX (Figure 1F), indicating that DJ-1 was recruited to DSB sites. The interaction of DJ-1 with γH2AX induced by ETO was further validated by in situ proximity ligation assay (PLA), which allows straightforward visualization and quantification of in situ interactions (protein pairs less than 40 nm away from each other) in fixed cells [36] (Figure 1G). To further prove the presence of DJ-1 at DNA DSBs, we performed immunostaining of another two DSB markers, 53BP1 and phosphorylated ATM (p-ATM) [37], with DJ-1, respectively. The results demonstrated that DJ-1 nuclear foci colocalized with both 53BP and p-ATM foci after DNA damage stimulation (Supplementary Figure S1C,D). Furthermore, we also performed biochemical fractionation to demonstrate the enrichment of DJ-1 in the chromatin fraction after ETO exposure (Supplementary Figure S1E).

### 2.2. DJ-1 Enhances DNA Damage Repair

**Figure 2:**
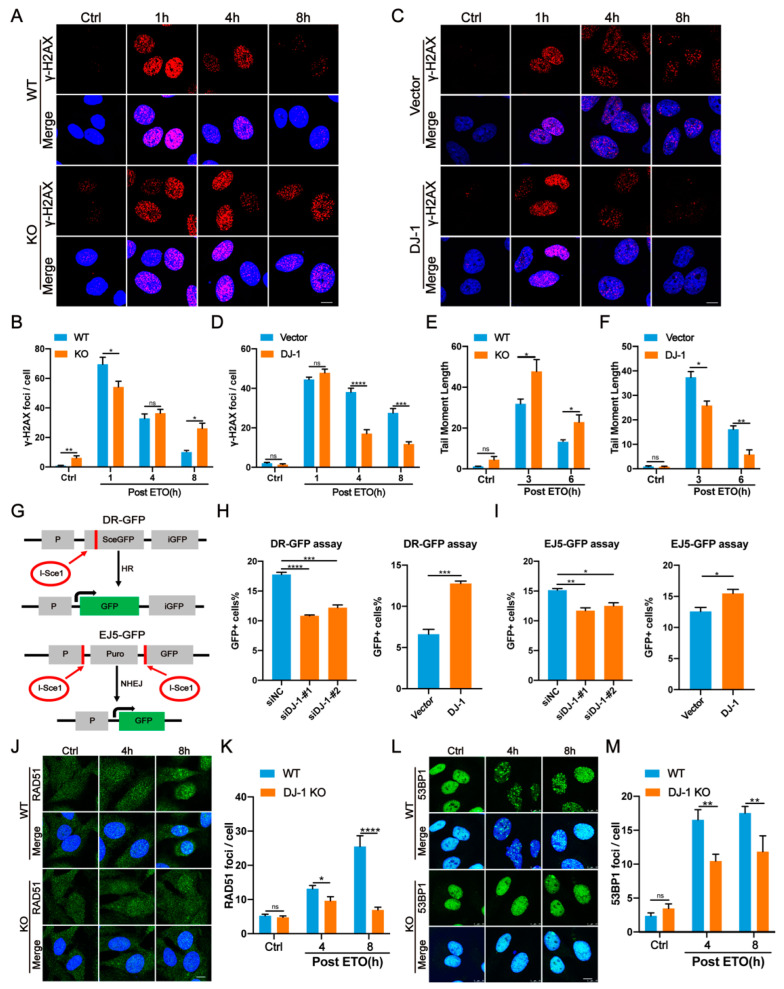
DJ-1 promotes DSB repair. (**A**,**B**) Representative immunofluorescence images and quantification of γ-H2AX foci in DJ-1 KO and WT U2OS cells at the indicated times after ETO treatment. Cells were treated with ETO (10 µM, 1 h) and were allowed to recover in an ETO-free medium. Scale bar, 10 μm. Data are presented as the mean ± SEM, n > 150 cells. *, *p* < 0.05; **, *p* < 0.01; ns, nonsignificant (two-tailed *t*-test). (**C**,**D**) Representative immunofluorescence images and the quantification of γ-H2AX foci in WT and the stable expression of DJ-1 U2OS cells at the indicated times after ETO treatment. Scale bar, 10 μm. Data are presented as the mean ± SEM, n > 150 cells. ***, *p* < 0.001; ****, *p* < 0.0001; ns, nonsignificant (two-tailed *t*-test). (**E**,**F**) Quantification of tail moments in DJ-1 KO (**E**) or stable expression of DJ-1 (**F**) U2OS cells treated with ETO (10 µM, 1 h) as determined by a neutral comet assay. Data are presented as the mean ± SEM, n > 100 cells. *, *p* < 0.05; **, *p* < 0.01; ns, nonsignificant (two-tailed *t*-test). (**G**) Schematics of HR and NHEJ reporter assays. (**H**,**I**) Quantification of DJ-1 knockdown and overexpression on HR (**H**) and NHEJ (**I**) in HEK293T cells. HEK293T cells transfected with the indicated siRNA or plasmids were subjected to DR-GFP assay or EJ5-GFP assay analyzed by flow cytometry. Data are presented as the mean ± SEM, n = 3. *, *p* < 0.05; **, *p* < 0.01; ***, *p* < 0.001; ****, *p* < 0.0001 (two-tailed *t*-test). (**J**,**K**) Representative immunofluorescence images and quantification of RAD51 foci in DJ-1 KO and WT U2OS cells treated with ETO (10 µM, 1 h) at the indicated time points. Scale bar, 10 μm. Data are presented as the mean ± SEM, n > 150 cells. *, *p* < 0.05; ****, *p* < 0.0001; ns, nonsignificant (two-tailed *t*-test). (**L**,**M**) Representative immunofluorescence images and quantification of 53BP1 foci in DJ-1 KO and WT U2OS cells treated with ETO (10 µM, 1 h) at the indicated time points. Scale bar, 10 μm. Data are presented as the mean ± SEM, n > 150 cells. **, *p* < 0.01; ns, nonsignificant (two-tailed *t*-test).

To study the role of DJ-1 in regulating DNA repair, we constructed human U2OS and M17 cells in which the DJ-1 gene was knocked out by the CRISPR/ Cas9 gene-editing technique. We first assessed the level of γH2AX, a well-known marker for DNA DSBs [32], in both cell types. Western blot analysis confirmed the accumulation of γH2AX both in U2OS and M17 DJ-1 KO cells (Supplementary Figure S2A,B). Consistently, immunofluorescence images showed significantly increased formation of γH2AX foci in DJ-1 KO cells (Figure 2A,B and Supplementary Figure S2C), suggesting an increased amount of DNA damage in DJ-1 defective models. In contrast, re-expression in DJ-1 KO cells with exogenous DJ-1 significantly reduced the number of γH2AX foci (Supplementary Figure S2D). In addition, the activation of DDR signaling, as reflected by p-ATM levels, was also observed in DJ-1 KO cells (Supplementary Figure S2E).

To investigate whether loss of DJ-1 affects DNA repair kinetics, the time course of the change in γH2AX foci in ETO-treated U2OS cells was assessed by immunofluorescence. The number of γH2AX foci were counted at 1, 4, and 8 h following exposure to ETO. Compared to WT, DJ-1 depletion resulted in a significantly delayed clearance of γH2AX foci after 8 h of recovery post-ETO treatment (Figure 2A,B). Interestingly, we also observed a reduction in γH2AX foci formation in DJ-1 KO U2OS cells 1 h after ETO treatment compared to controls (Figure 2A,B), suggesting that DJ-1 may affect the initial sensing of DSBs and H2AX phosphorylation. As DNA damage elevated endogenous DJ-1 levels, we also measured the formation and resolution of ETO-induced γH2AX foci in cells stably overexpressing DJ-1. We observed a significantly faster clearance of γH2AX foci, starting 4 h after the initial insult, in U2OS cells overexpressing DJ-1 (Figure 2C,D). Furthermore, these were also confirmed in treatments with CPT and MMS. Namely, DJ-1 overexpression cleared γH2AX foci at a more rapid kinetic rate (Supplementary Figure S3A,B), whereas DJ-1 KO resulted in γH2AX foci accumulation (Supplementary Figure S3C,D). 

To further confirm the role of DJ-1 in repairing DSBs, we performed neutral comet assays that were conducted at 3 h and 6 h post-ETO, and calculated tail moments to assess the levels of DNA breaks. We observed that a fraction of DJ-1 KO U2OS cells showed comet tails even without ETO treatment (Figure 2E and Supplementary Figure S3E), although the difference was not statistically significant. After ETO withdrawal and recovery for the indicated time, the tail moments were significantly reduced in WT cells, whereas DJ-1 KO cells continued to have longer comet tails (Figure 2E and Supplementary Figure S3E). In contrast, the stable expression of DJ-1 reduced the tail moments of U2OS cells at 3 h and 6 h post-ETO (Figure 2F and Supplementary Figure S3F). These results indicated that DJ-1 deficiency could cause a substantial reduction in DSB repair efficiency. We also investigated whether DJ-1 deficiency increased the susceptibility of cells to DNA-damaging agents. Compared with WT, DJ-1 KO U2OS cells were more vulnerable to genotoxic stress evaluated via CCK-8 assays (Supplementary Figure S4A), whereas stable expression of DJ-1 significantly protected cells against genotoxic agents and facilitated cell survival (Supplementary Figure S4B). Taken together, these findings demonstrate that DJ-1 deficiency impairs, while DJ-1 overexpression increases the DNA damage repair ability.

### 2.3. DJ-1 Promotes Both HR- and NHEJ-Mediated DNA Repair

For DSB repair, there are two major pathways: homologous recombination (HR) and nonhomologous end joining (NHEJ) [38]. The role of DJ-1 on HR was examined by a well-established HR reporter plasmid DR-GFP, which contains two nonfunctional GFP genes [39,40] (Figure 2G). The first GFP gene is disrupted by the insertion of an I-Sce1 restriction enzyme site [39,40]. GFP expression is restored when I-Sce1-induced DSBs are repaired by HR, which can be monitored by flow cytometry [39,40]. In DJ-1 knockdown cells, HR activity was lower than that in controls, whereas DJ-1 overexpression increased HR activity (Figure 2H and Supplementary Figure S5). Meanwhile, DJ-1 depletion did not result in any obvious changes in cell cycle profile (Supplementary Figure S6). In addition, DJ-1’s involvement in HR was further investigated by monitoring the formation of RAD51 foci, an indicator of HR repair [41], by immunofluorescence in a time-course experiment. After ETO exposure, DJ-1 deficiency resulted in a marked reduction in RAD51 foci formation throughout the repair process (Figure 2J,K). Conversely, stable expression of DJ-1 led to a significant rise in RAD51 foci formation (Supplementary Figure S7A). To examine whether DJ-1 facilitates NHEJ, we performed assays using the EJ5-GFP reporter, which contains a promoter separated from a silent GFP gene by a puromycin gene flanked by two I-Sce1 sites [39,40] (Figure 2G). The expression of GFP is restored after NHEJ repairs I-Sce1-induced DSBs [39,40]. It was found that the overexpression of DJ-1 enhanced, whereas the knockdown of DJ-1 reduced, NHEJ-mediated DSB repair (Figure 2I). We also longitudinally monitored the formation of 53BP1 foci, which facilitates NHEJ repair of DSBs [41], and observed a significantly reduced number of 53BP1 foci in DJ-1 KO cells after ETO treatment, compared to WT (Figure 2L,M). In contrast, 53BP1 foci were significantly increased in stable expression of DJ-1 cells compared to controls, supporting the role of DJ-1 in promoting NHEJ (Supplementary Figure S7B). 

### 2.4. DJ-1 Interacts with PARP1

**Figure 3:**
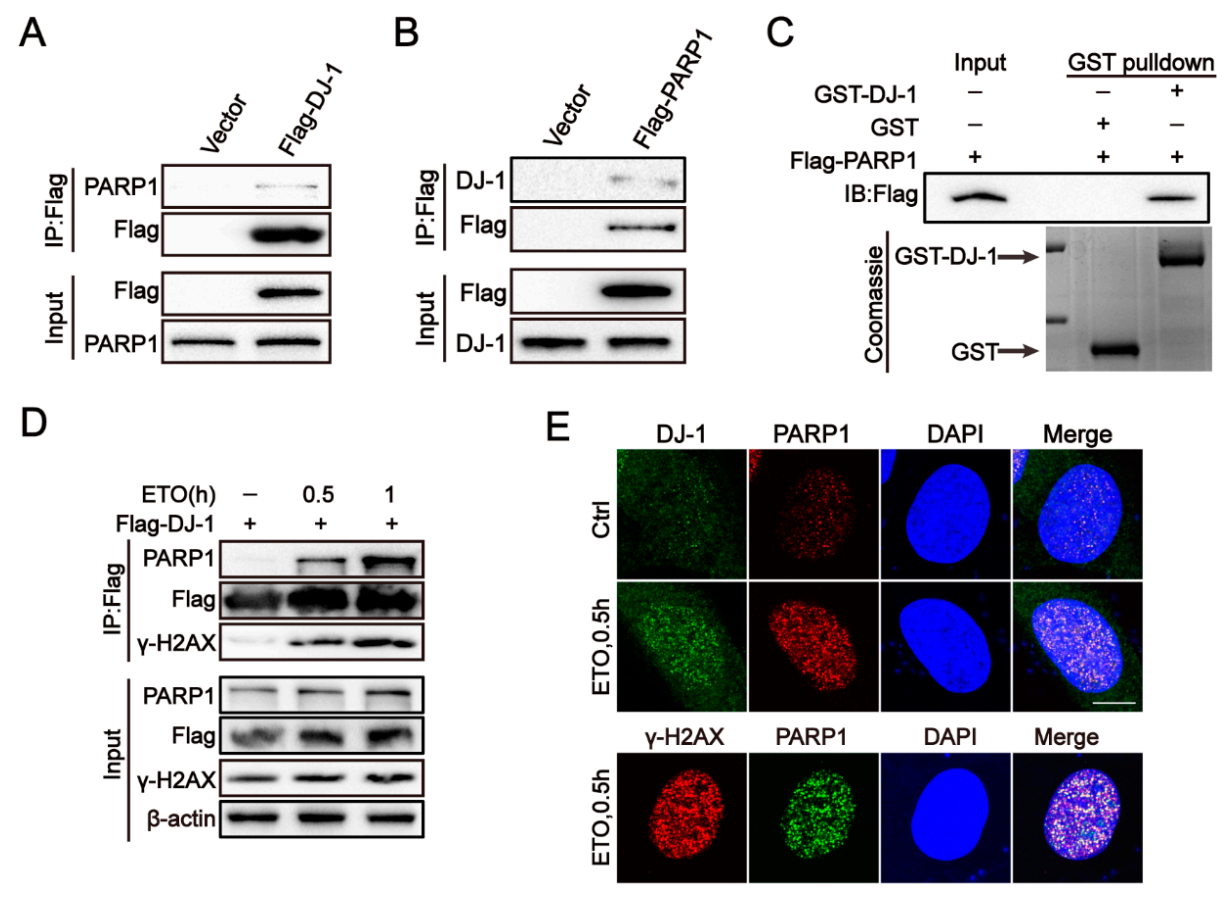
DJ-1 interacts with PARP1. (**A**,**B**) Representative co-immunoprecipitation (Co-IP) analysis of DJ-1 and PARP1 in HEK293T cells. Exogenous Co-IP were performed using the indicated antibodies. (**C**) Purified GST-DJ-1 or GST protein was incubated Flag-PARP1 expressed from HEK293T cells and blotted with antibody to Flag. GST or GST-DJ-1 was detected by coomassie staining (bottom). (**D**) Representative Co-IP analysis of DJ-1 and PARP1 after ETO (10 µM) treatment for the indicated times in HEK293T cells. (**E**) Representative immunostaining of DJ-1, PARP1, and γ-H2AX following ETO (10 µM) treatment in U2OS cells at the indicated times. Scale bar, 10 μm. n = 3.

Next, we investigated the mechanistic basis by which DJ-1 facilitates the DSB repair. PARP1 is a critical DNA repair enzyme involved in several DNA repair pathways, including HR, NHEJ, NER, base excision repair (BER), and mismatch repair (MMR) [42]. Recently, a mass spectrometry-based screen suggested an association between PARP-1 and DJ-1 [43]. Thus, the Co-IP experiment was performed, and as expected, the interaction between DJ-1 and PARP1 was verified in HEK293T cells ectopically expressing DJ-1, as well as endogenous proteins in 293T cells (Figure 3A,B and Supplementary Figure S8A). A direct interaction between DJ-1 and PARP1 was further verified by GST pull-down assays (Figure 3C). Importantly, a significantly enhanced interaction between DJ-1 and PARP1 was found when treated with ETO (Figure 3D), which was resistant to DNA degrading enzyme Benzonase (Supplementary Figure S8B), indicating that the increasing interaction was direct, rather than mediated by DNA. Then the interaction between DJ-1 and PARP1 in U2OS cells was further confirmed by PLA, and the PLA signals were obviously increased in the presence of ETO (Supplementary Figure S8C). In the meantime, immunostaining images demonstrated that the DJ-1 and PARP1 colocalization was located in the nucleus and was enhanced after ETO treatment in U2OS cells (Figure 3E). Additionally, after treatment with ETO, a substantial fraction of PARP1 colocalized with γH2AX foci, suggesting that the interaction between DJ-1 and PARP1 is located at sites of DSBs, which is further confirmed by the presence of γH2AX in the Co-IP complex of DJ-1 and PARP1 (Figure 3D).

### 2.5. DJ-1 Directly Regulates PARP1 Activity

**Figure 4:**
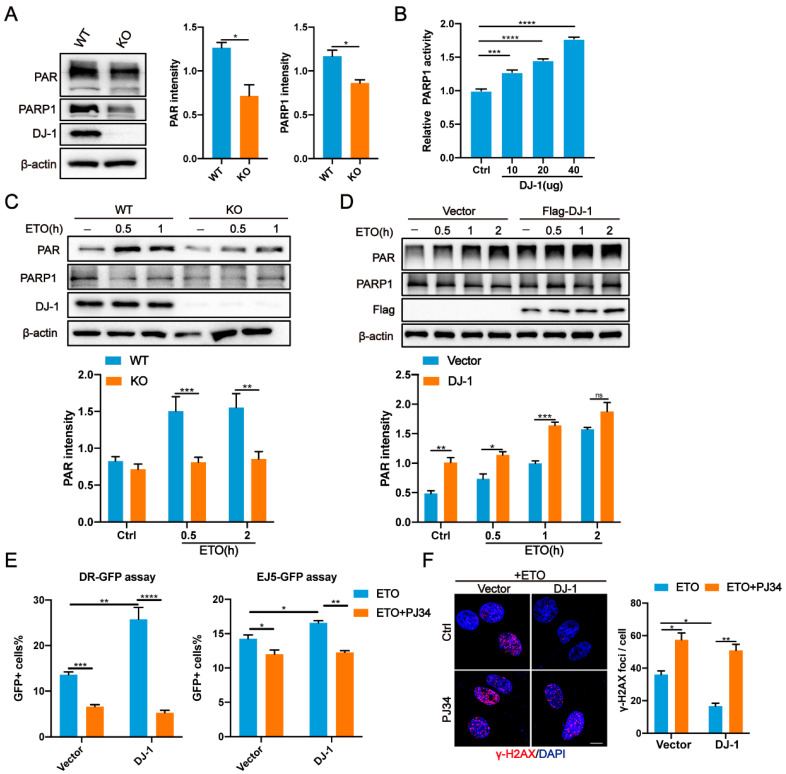
DJ-1 stimulates PARP1 activity. (**A**) Representative western blot and quantification of PAR and PARP1 proteins in DJ-1 KO and WT U2OS cells. Data are presented as the mean ± SD, n = 3. *, *p* < 0.05 (two-tailed *t*-test). (**B**) In vitro PARP1 enzymatic activity assay in the presence of recombinant DJ-1 protein. Data are presented as the mean ± SEM, n = 3. ***, *p* < 0.001; ****, *p* < 0.0001 (two-tailed *t*-test). (**C**) Representative western blot and quantification of PAR in WT and DJ-1 KO U2OS cells treated with ETO (10 µM) at the indicated time points. Data are presented as the mean ± SD, n = 3. **, *p* < 0.01; ***, *p* < 0.001 (two-tailed *t*-test). (**D**) Representative western blot and quantification of PAR in U2OS cells expressing Flag-DJ-1 following ETO (10 µM) at the indicated time points. Data are presented as the mean ± SD, n = 3. *, *p* < 0.05; **, *p* < 0.01; ***, *p* < 0.001; ns, nonsignificant (two-tailed *t*-test). (**E**) Quantification of NHEJ and HR in HEK293T cells expressing Flag-DJ-1 in the presence and absence of PJ34 (10 µM). Data are presented as the mean ± SEM, n = 3. *, *p* < 0.05; **, *p* < 0.01; ***, *p* < 0.001; ****, *p* < 0.0001 (two-tailed *t*-test). (**F**) Representative immunofluorescence images and quantification of γ-H2AX in WT and stable expression of DJ-1 U2OS cells. U2OS were treated with ETO (10 µM, 1 h) in the presence and absence of PJ34 (10 µM) and were allowed to recover for 8 h. Scale bar, 10 μm. Data are presented as the mean ± SEM, n > 150 cells. *, *p* < 0.05; **, *p* < 0.01 (two-tailed *t*-test).

As we had demonstrated that DJ-1 binds to PARP1, this raised the possibility that DJ-1 could regulate PARP1 activity. We first examined the levels of PAR [poly (ADP-ribose)], an indicator of PARP1 activity [31,44]. Western blot analysis confirmed the decreased PAR both in U2OS and M17 DJ-1 KO cells (Figure 4A and Supplementary Figure S9A). Of note, decreased levels of PARP1 were also seen in DJ-1 KO cells. However, PARP1 mRNA levels remained unchanged (Supplementary Figure S9B), indicating that DJ-1 regulated PARP1 at the protein level. To determine whether DJ-1 directly activates PARP1 by binding to PARP1, we conducted PARP1 activity assay in the presence of recombinant DJ-1 protein and observed concentration-dependent activation of PARP1 by DJ-1 in vitro (Figure 4B). Moreover, in the presence of genotoxic agents, DJ-1 KO U2OS cells had decreased levels of PAR production compared to WT (Figure 4C and Supplementary Figure S9C). In contrast, DJ-1 overexpression enhanced PAR levels before and after exposure to genotoxic agents without affecting PARP1 expression (Figure 4D and Supplementary Figure S9D,E).

To determine whether DJ-1-mediated DNA repair is dependent on PARP1, we inhibited PARP1 activity using PJ34 [45]. As expected, inhibition of PARP1 not only significantly attenuated the increased PAR levels due to DJ-1 overexpression but also reduced the increased NHEJ and HR activity (Figure 4E and Supplementary Figure S10A). Moreover, the faster clearance of ETO-induced γH2AX foci in U2OS cells stably expressing DJ-1 was effectively counteracted by PJ34 (Figure 4F). Notably, PARP1 inhibition led to similar DNA damage and repair defects as DJ-1 deficiency (Figure 4E,F and Supplementary Figure S10B). Taken together, these data support the notion that DJ-1 interacts with PARP1 and activates its enzymatic activity in response to DNA damage, contributing to the observed role of DJ-1 in promoting DNA repair.

### 2.6. DNA Damage Accumulation and Defective PARP1 Activity in DJ-1 Mutant Fibroblasts

**Figure 5:**
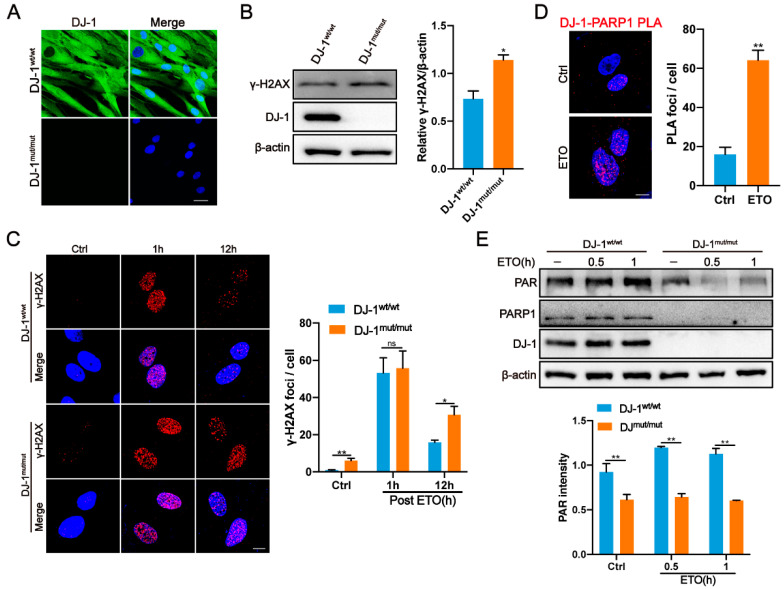
DJ-1 deficiency leads to DNA damage accumulation and defective PARP1 activity in PD fibroblasts. (**A**) Representative immunofluorescence images of DJ-1 in WT and DJ-1 mutant fibroblasts. Scale bar, 10 μm. (**B**) Representative western blot and quantification of γ-H2AX in WT and DJ-1 mutant fibroblasts. Data are presented as the mean ± SD, n = 3. *, *p* < 0.05 (two-tailed *t*-test). (**C**) Representative immunofluorescence images and quantification of γ-H2AX foci in WT and DJ-1 mutant fibroblasts at the indicated times after ETO treatment. Cells were treated with ETO (10 µM, 1 h) and were allowed to recover in an ETO-free medium. Scale bar, 10 μm. Data are presented as the mean ± SEM, n > 150 cells. *, *p* < 0.05; **, *p* < 0.01; ns, nonsignificant (two-tailed *t*-test). (**D**) Representative PLA signals between DJ-1 and PARP1 in fibroblasts following ETO (10 µM, 1 h) treatment. Quantification of average PLA signals are shown on the right. Scale bar, 10μm. Data are presented as the mean ± SEM, n > 100 cells. **, *p* < 0.01 (two-tailed *t*-test). (**E**) Representative western blot and quantification of PAR in WT and DJ-1 mutant fibroblasts treated with ETO (10 µM) at the indicated time points. Data are presented as the mean ± SD, n = 3. **, *p* < 0.01 (two-tailed *t*-test).

Recently, Lin et al. identified a novel DJ-1 homozygous mutation (c.390delA, p.D131Tfs∗3) in a Chinese consanguineous family [46]. The proband of this family manifested parkinsonism at 22 years old [46]. Human skin fibroblasts derived from this early onset PD patient carrying the DJ-1 mutation were obtained for the subsequent analysis. Consistent with the data from Lin et al., the western blot analysis and immunofluorescence revealed that DJ-1 with c.390delA, p.D131Tfs*3 was almost fully degraded compared to controls (Figure 5A,B). 

We next explored whether the DJ-1-mediated regulation of DNA repair also occurred in PD patient cells. Indeed, untreated DJ-1 mutant fibroblasts displayed elevated levels of γH2AX accumulation and foci formation compared to controls (Figure 5B,C). Moreover, DJ-1 deficiency resulted in a significantly delayed clearance of γH2AX foci after 12 h of recovery post-ETO treatment, suggesting that DJ-1 mutant fibroblasts are also deficient in DSB repair (Figure 5C). PLA also confirmed the interaction between DJ-1 and PARP1 in skin fibroblasts, and the association of both increased after DNA damage (Figure 5D). Consistent with the results observed in DJ-1 KO cells, PARP1 activity was significantly reduced in DJ-1 mutant fibroblasts with or without ETO treatment compared to controls (Figure 5E). Overall, these results demonstrate a defect in DNA repair in PD patients’ fibroblasts due to DJ-1 loss of function mutation.

## 3. Discussion

PD is the second most common neurodegenerative disease with no clear pathogenesis [47]. Recent advances have demonstrated that DNA damage and repair deficiency play an important role in the pathophysiology of PD [9,10]. However, the mechanism underlying DNA repair abnormalities in PD remains unclear. 

DJ-1 is a neuroprotective protein [23]. Since the discovery of loss-of-function mutations in DJ-1 causing PD in 2003, research has mainly focused on the role of DJ-1 in the cytoplasm, especially in mitochondria [48,49,50]. In fact, DJ-1 is also distributed in the nucleus, and previous studies have revealed that DJ-1 can also translocate into the nucleus in response to various stress conditions, but little is known about its functions [23,26]. It was recently reported that DNA damage accumulates in DJ-1 defective animal models, and that DJ-1 deficiency increases the susceptibility to genomic toxicity [27,28,29], providing the premise for this study to explore the role of DJ-1 in DNA repair. Here, we demonstrated, for the first time, that DJ-1 is also a DDR protein. The expression of DJ-1 can be induced by different genotoxic drugs, and this phenomenon exists in a variety of cell types. Additionally, a significant intranuclear aggregation of DJ-1 was observed after DNA damage. More importantly, similar to most DNA repair proteins, DJ-1 is able to form foci in the nucleus and can be recruited to the sites of DNA damage [34], suggesting a direct action of DJ-1 in DNA repair.

Consistent with previous findings, we found that γH2AX foci as well as protein expression were significantly upregulated in DJ-1 defective cell models, indicating the existence of DNA damage [29]. Moreover, after DNA damage stimulation, the clearance efficiency of γH2AX foci in DJ-1 KO cells was significantly reduced compared to controls, suggesting that DJ-1 deficiency affects the DNA repair process, which in turn leads to DNA damage accumulation. The comet assays further confirmed the facilitating effect of DJ-1 on the DNA repair process. Surprisingly, we also showed that DJ-1 deficiency impairs the initial formation of γH2AX foci after DNA damage. It has been well reported that the formation of γH2AX foci is a marker of early DDR, and that γH2AX can amplify DDR signaling by recruiting DNA repair proteins [51,52]. Thus, our findings suggest that DJ-1 may play a role in the early stage of DDR. Interestingly, similar findings have been reported for TDP43 and FUS, implying their functions in the initiation of DNA repair [32,35].

It is well known that DSB repair consists of two main pathways, NHEJ and HR [42,53]. HR is thought to occur mainly during the S and G2 phases of the cell cycle and is considered to be an error-free DNA repair mechanism because it uses the intact DNA sequences on homologous sister chromatids as templates [53]. In contrast, NHEJ occurs throughout the cell cycle [53]. NHEJ is an error-prone repair mechanism because there is no complete template for repair, which can lead to insertions, deletions, or chromosomal translocations [53]. Since neurons are post-mitotic and, therefore, nonreplicative, it was thought that HR did not occur in neurons [9]. However, recent studies have shown that neurons can use mRNA as a template for HR repair, implying that this mechanism exists in neurons [10]. Here, by using a well-established reporting system for DSB repair, we confirmed that DJ-1 can affect both HR and NHEJ. Consistently, we observed that the recruitment of HR and NHEJ repair factors was inhibited by DJ-1 deficiency. 

Mechanistically, we demonstrated that DJ-1 directly interacted with PARP1, and that this interaction is important for PARP1 activation. PARP1 is a nuclear enzyme activated by DNA damage [42,54]. Activated PARP1 has been implicated in different DNA repair pathways, including DSB repair [42,54]. It has been reported that PARP1 can stimulate classical NHEJ through both a DNA-PK-dependent and DNA-PK-independent manner and promote alternative NHEJ by recruiting MRE11 [42]. In HR, PARP1 activity is crucial for the control and recruitment of HR proteins [42]. In addition, PARP1 is important for the early DDR signaling initiated by ATM, as PARP1 inhibition or deficiency leads to delayed activation of DDR proteins, such as γH2AX, p53, and SMC1 [42]. Corresponding to the increased binding of DJ-1 to PARP1 and upregulated PAR levels after DNA damage, DJ-1 overexpression promoted the recruitment of DNA repair factors, thereby accelerating DNA repair. In contrast, DJ-1 deficiency compromised PARP1 activity and disrupted the initial DSB signaling and subsequent DNA repair. These observations are consistent with the elucidated roles of PARP1 in the early DDR and DSB repair, and provide mechanistic insights into the function of DJ-1 in these processes [42]. However, it remains difficult to understand how DJ-1 stimulates PARP1 enzyme activity, although DJ-1 has recently been defined as a deglycase, suggesting that it possesses enzymatic activity [55]. Structural studies will be very valuable to elucidate these distinct properties of DJ-1. Additionally, our data do not rule out the possibility that DJ-1 has additional interaction partners within and outside of the DDR. In fact, Kazuko et al. showed that DJ-1 directly binds to SIRT6, which similarly binds and stimulates PARP1 activity to promote DSB repair [45,56]. Recently, Onn et al. identified SIRT6 as a DSB sensor capable of binding to DSBs and initiating DDR [57]. Thus, it will be interesting to further examine if there is a direct crosstalk between DJ-1 and SIRT6 in the regulation of PARP1 and the initial DSB signaling events.

This study has several limitations. First, we explored the role of DJ-1 in DNA repair mainly in proliferating cells. While we also confirmed the DNA damage phenotype in a DA neuronal cell line (M17 cells), it remains to be further verified whether the regulatory effect of DJ-1 on DNA repair is also available in primary neurons. Second, we observed that DJ-1 is recruited to DNA damage sites, yet the specific molecular mechanism that mediates DJ-1 recruitment remains unclear. Although there is an interaction between DJ-1 and PARP1, PARP1 appears to have no effect on DJ-1 recruitment. Finally, we only demonstrated that DJ-1 directly stimulates PARP1, but it is unclear how this enhancement works at the molecular level.

In summary, our work has demonstrated that DJ-1, as a DDR protein, is important for effective DSB repair. DJ-1 maintains genomic stability together with PARP1, and the impairment of this interaction may contribute to impaired DNA repair and DNA damage accumulation and eventually neurodegeneration. Thus, our data offer insight into the molecular mechanisms underlying DNA repair defects in PD, as well as the potential pathogenic mechanisms of DJ-1 deficiency in PD.

## 4. Materials and Methods

### 4.1. Cell Lines and Human Fibroblast Cultures

Human U2OS, M17, HEK293T cells and primary skin fibroblasts were grown in Dulbecco’s Modified Eagle Medium (DMEM) containing 10% fetal bovine serum (FBS) and 1% penicillin/streptomycin. Skin fibroblasts containing DJ-1 mutation, a homozygous mutation (c.390delA) in exon 6, were a kind gift from Professor Zhi-Ying Wu at Zhejiang University Medical School. Primary fibroblasts were established from skin biopsies obtained from relatives and healthy controls. 

### 4.2. Antibodies and Chemical Reagents

The following antibodies were purchased from Cell Signaling Technology: anti-DJ-1 (#5933), anti-γH2AX (#9718), anti-Flag (#14793), anti-53BP1 (#88439), anti-PARP1 (#9532), anti-RAD51 (#8875), anti-PAR (#83732), and anti-histone H3 (#4499). Anti-phospho S1981-ATM (ab36810), anti-γH2AX (ab22551), and anti-DJ-1 (ab11251) were purchased from Abcam. Anti-β-actin (AC026) was purchased from Abclonal. Horseradish peroxidase (HRP) conjugated goat anti-rabbit and HRP conjugated goat anti-mouse antibodies were obtained from HUABIO. Alexa Fluor-488 conjugated anti-rabbit (A21206) and Alexa Fluor-555 conjugated anti-mouse (A21424) antibodies were obtained from Thermo Fisher. Etoposide (S1225), camptothecin (S1288), and PJ34 (S7300) were purchased from Selleck. Methyl methanesulfonate (129925) and Benzonase (70664) were obtained from Sigma-Aldrich.

### 4.3. Plasmids, siRNA Transfection, and Lentiviral Infection

DJ-1 and PARP1 plasmids were synthesized by Gene Create (Wuhan, China). All construct sequences were verified using DNA sequencing. Lipofectamine^®^ 3000 (Invitrogen, L3000015) was used to perform transient transfection of plasmid DNA according to the protocol. siRNA transfection was performed using Lipofectamine RNA iMAX (Invitrogen, 13778030) following the protocol. DJ-1 siRNAs were purchased from GenePharma (Shanghai, China). The target sequences of the siRNAs are available in Supplementary Table S1. As previously described, DJ-1 KO cells were generated using CRISPR-Cas9 technology [6]. M17 and U2OS cells were transfected with an expression vector containing DJ-1 single-guide RNA using lentiviruses. The cells were then cultured for 48 h in the presence of puromycin (1 μg/mL). After serial dilution into 96-well plates, the single clones obtained were subjected to western blot to examine the efficiency of DJ-1 knockout.

### 4.4. Western Blot

Proteins were lysed on ice for 30 min using RIPA lysis buffer (Beyotime, P0013B, China) with a protease inhibitor cocktail (Invitrogen, 78430, USA) and PMSF (Beyotime, ST506). Lysates were subjected to sonication, and then centrifuged at 12,000 rpm for 20 min at 4 °C. The supernatants were collected. Protein concentration was measured using a BCA protein assay kit (Invitrogen, 23225). The protein extracts were supplemented with 5× sodium dodecyl-sulfate (SDS) loading buffer and boiled for 10 min. Proteins were separated by sodium dodecyl sulfate-polyacrylamide gel electrophoresis (SDS-PAGE) and then transferred to polyvinylidene difluoride (PVDF) membranes (Merck Millipore, GVWP04700, Germany), which were blocked in 5% milk for 1 h at room temperature. The membranes were incubated with primary antibodies overnight at 4 °C and then washed. The secondary antibodies were added to membrane for 1 h at room temperature and then washed extensively. Subsequently, the immunoreactive bands were detected using a chemiluminescence reagent (Invitrogen, 34577).

### 4.5. Co-Immunoprecipitation (Co-IP)

Co-IP was conducted using the Dynabeads™ Protein G Immunoprecipitation Kit (Invitrogen, 10007D). Cells were treated as stated and lysed with 500 µL of IP lysis buffer (Beyotime, P0013) on ice. The lysates were centrifuged at 4 °C for 30 min at 12,000 rpm. Next, 2 μg of primary antibodies or normal IgG was incubated with 50 μL magnetic beads at room temperature for 60 min. Proteins in the lysates were incubated with indicated antibody coated beads at 4 °C overnight. The mixture of proteins and beads was washed with the wash buffer. Then, the beads were boiled in 2× SDS loading buffer for western blot.

### 4.6. GST Pull-Down Assay

The purified GST-DJ-1 protein was purchased from MedChemExpress (MCE, HY-P71625). The purity of the enriched proteins was determined by SDS-PAGE analysis and Coomassie staining. GST pull-down was performed using the Pierce GST Protein Interaction Pull-Down Kit (Thermo Scientific, 21516, USA) according to the protocol.

### 4.7. Immunofluorescence

Cells were seeded and cultured on glass coverslips in a 24-well plate. After treatment, cells were fixed in 4% paraformaldehyde for 30 min at room temperature. The cells were then permeabilized with 0.5% Triton X-100 for 15 min. After that, slides were blocked in 5% BSA for 1 h and then incubated with primary antibodies in 1% BSA overnight at 4 °C, followed by fluorescent secondary antibodies at room temperature for 1 h. Cell nuclei were stained with DAPI (Beyotime, C1006). Fluorescence images were captured using a Leica TSC SP8 confocal microscope. 

### 4.8. Proximity Ligation Assays (PLA)

In situ PLA were conducted using Duolink^®^ reagents (Sigma-Aldrich, DUO92101, USA) following the protocol. Immunofluorescence staining protocol was performed until primary antibody incubation. Secondary antibodies were coupled to PLA probes and ligated, followed by rolling circle amplification. Colocalization of both proteins leads to the appearance of the red PLA signal, which was visualized by Leica TSC SP8 confocal microscopy.

### 4.9. Cell Viability Assay

The Cell Counting Kit-8 assay (APExBIO, K1018) was used to measure cell viability. Cells were seeded in 96-well plates and after treatment, 100 µL medium containing 10 µL CCK-8 solution was added to each well. After incubation for 1–3 h, the absorbance of each well was measured at 450 nm using a spectrophotometer (Molecular Devices SpectraMax 190, Sunnyvale, CA, USA).

### 4.10. Real-Time Quantitative PCR (qPCR)

The total RNA was isolated using TRIzol reagent (Invitrogen, 15596018). Synthesis of cDNA was performed using the Evo M-MLV RT Premix for qPCR (Accurate Biotechnology, AG11706, China). qPCR was further conducted using the SYBR^®^ Green Premix Pro Taq HS qPCR Kit (Accurate Biotechnology, AG11701). All qPCR data were normalized to GAPDH using the 2^−ΔΔCt^ method. The primer sequences are shown in Supplementary Table S1.

### 4.11. Comet Assay 

The comet assay kit (Abcam, ab238544, UK) was used to perform the comet assay. Cells were first mixed with agarose and then laid on comet slides. The slides were soaked in prechilled lysis solution at 4 °C for 60 min, followed by alkaline unwinding solution for 30 min. Then, the slides were subjected to electrophoresis in the dark for 30 min at 20 V. Nuclear DNA was stained with SYBR Green dye for 15 min and then visualized under a fluorescence microscope. The tail moment was calculated using Open Comet.

### 4.12. HR and NHEJ Assays 

HR and NHEJ assays were performed according to previous reports [38]. In brief, NHEJ reporter plasmid pimEJ5GFP (Addgene, #44026, USA) or HR reporter plasmid DR-GFP (Addgene, #26475) was transfected in HEK 293T cells. To check the effect of DJ-1 depletion on NHEJ and HR efficiency, cells were transfected with either DJ-1 siRNA or control. The pCBASceI plasmid (Addgene, #26477) was transfected into both control and DJ-1 siRNA-treated cells at 48 h post-transfection. After 24 h, cells were harvested and subjected to flow cytometry (Beckman CytoFLEX). To investigate the effect of DJ-1 overexpression on NHEJ and HR efficiency, cells were transfected with empty vector/ Flag-DJ-1 and pCBASceI plasmids. Cells were collected after 48 h, and the GFP signal was evaluated by flow cytometry.

### 4.13. Subcellular Fractionation

For the preparation of cytoplasmic and nuclear extracts, a nuclear extraction kit (Beyotime, P0028) was used according to the protocol. Chromatin was isolated using the chromatin extraction kit (Abcam, ab117152) following the protocol.

### 4.14. PARP1 Activity Assay

The PARP1 enzyme activity assay kit (Trevigen, # 4684-096-K, USA) was used to evaluate PARP1 activity. In brief, GST and GST-tagged recombinant DJ-1 were added to the assay mixture and reacted for 60 min. PARylation of PARP1 was calculated using a colorimetric assay and read at 450 nm using a spectrophotometer.

### 4.15. Cell Cycle Analysis

The cell cycle was determined by propidium iodide (PI) staining (Selleck, S6874, China). DJ-1 WT and KO cells were plated in six-well plates, after 48 h, cells were collected and fixed with 70% ethanol for 12 h. Then, cells were stained with PI for 30 min and subjected to flow cytometry.

### 4.16. Statistical Analyses

Data are presented as mean ± SD or SEM. Statistical analysis was performed using GraphPad Prism 7 software. For comparisons between two groups, we performed an unpaired, two-tailed Student’s *t*-test. For all analyses, statistical significance was defined as *p* < 0.05.

## Data Availability

All data supporting the findings from this study are available within the article and its supplementary information.

## Supplementary Materials

The following supporting information can be downloaded at: https://www.mdpi.com/article/10.3390/ijms24108651/s1.
