# Supplementary material for: Nuclear DJ-1 Regulates DNA Damage Repair via the Regulation of PARP1 Activity
Source: Int J Mol Sci. 2023 May 12;24(10):8651. doi: 10.3390/ijms24108651 (PMC10218208; doi:10.3390/ijms24108651)
Supplement: Supplementary file 1 [file ijms-24-08651-s001.zip › ijms-2294236-supplementary.pdf]

Supplementary Information

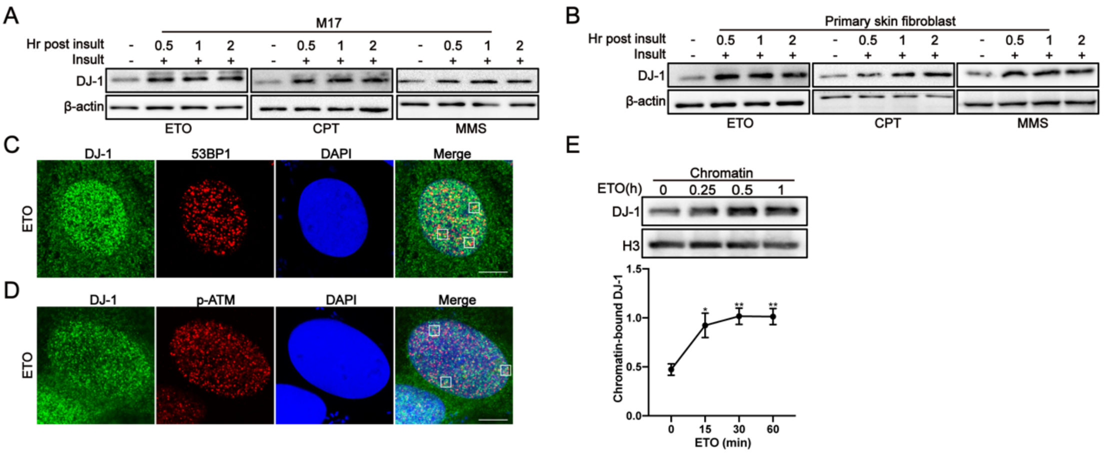

Figure S1. DJ-1 responds to DSBs. (A, B) Representative western blot of DJ-1 in M17 cells and primary human skin fibroblasts treated with ETO (10 μM), CPT (10 μM), and MMS (0.01%) at the indicated timepoints. n = 3. (C) Representative immunostaining of 53BP1 and DJ-1 following ETO (10 μM, 30 min) treatment in U2OS cells. Scale bar, 10 μm. n=3. (D) Representative immunostaining of p-S1981-ATM and DJ-1 following ETO (10 μM, 30 min) treatment in U2OS cells. Scale bar, 10 μm. n=3. (E) Representative western blot and quantification of DJ-1 in chromatin fraction following ETO (10 μM) treatment at the indicated timepoints in U2OS cells. Histone H3 was used as positive marker for chromatin fraction. Data are presented as the mean ± SD, n=3. *, P < 0.05; **, P < 0.01 (two tailed t-test).

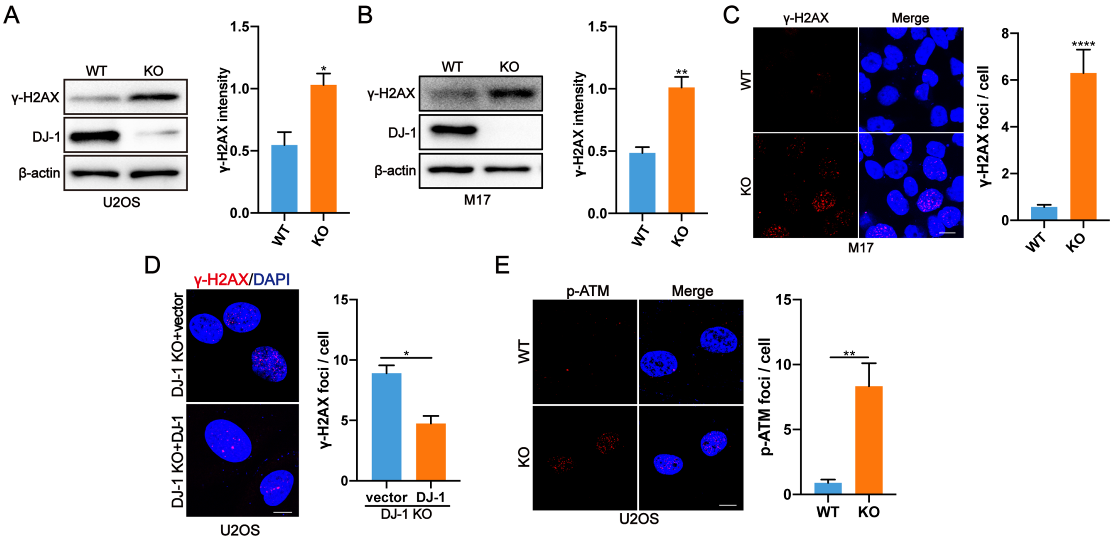

Figure S2. Loss of DJ-1 results in DNA damage accumulation and activation of DNA

damage response signaling. (A, B) Representative western blot and quantification of γ-H2AX in DJ-1 KO and WT U2OS (A) and M17 (B) cells. Data are presented as the mean ± SD, n = 3. *, $P < 0.05$; **, $P < 0.01$ (two tailed t-test). (C) Representative immunofluorescence images and quantification of γH2Ax foci in WT and DJ-1 KO M17 cells. Scale bar, 10 μm. Data are presented as the mean ± SEM, n > 150 cells. ****, $P < 0.0001$ (two tailed t-test). (D) Representative immunofluorescence images and quantification of γH2Ax foci in DJ-1 KO U2OS cells with and without DJ-1 stable expression. Scale bar, 10 μm. Data are presented as the mean ± SEM, n > 150 cells. *, $P < 0.05$ (two tailed t-test). (E) Representative immunofluorescence images and quantification of p-ATM-S1981 foci in WT and DJ-1 KO U2OS cells. Scale bar, 10 μm. Data are presented as the mean ± SEM, n > 150 cells. **, $P < 0.01$ (two tailed t-test).

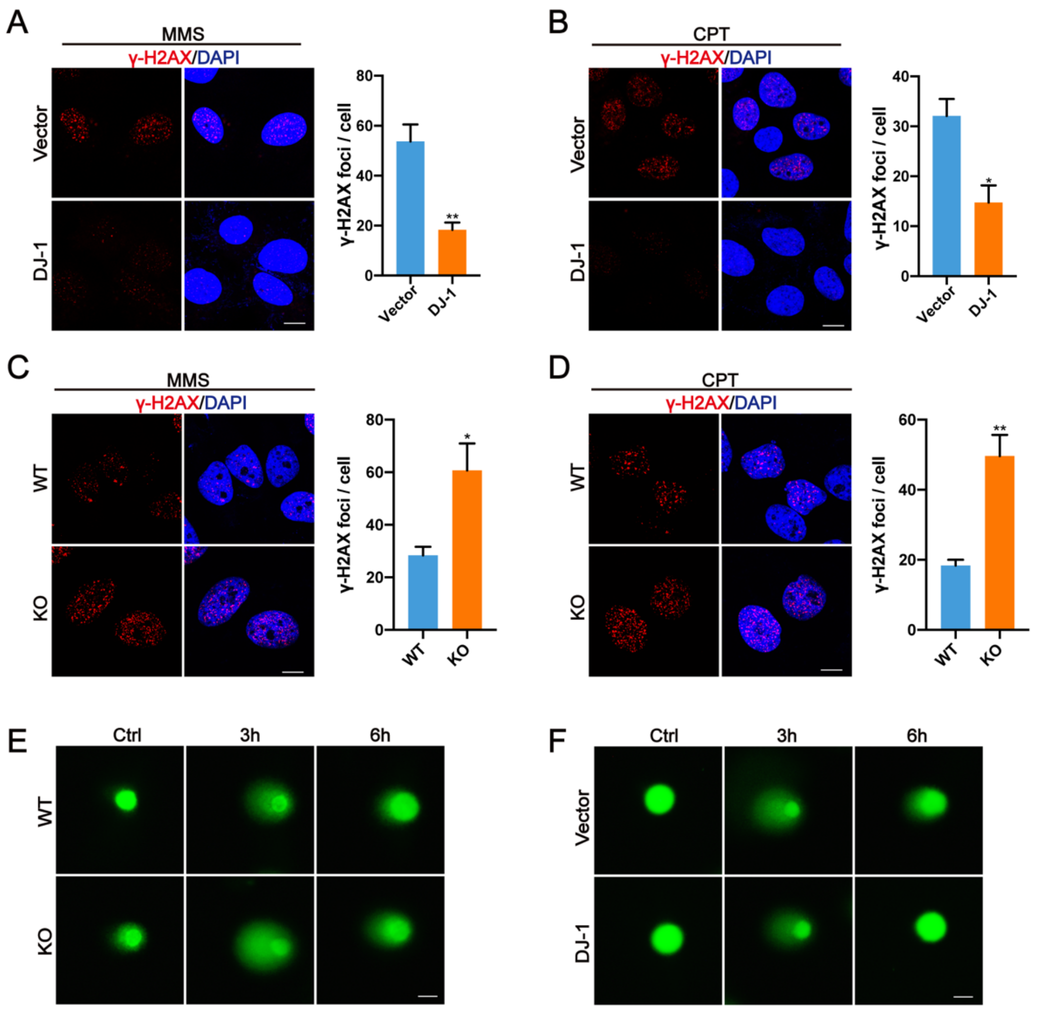

Figure S3. DJ-1 enhances DNA repair. (A, B) Representative immunofluorescence images and quantification of γ-H2AX foci in WT and stable expression of DJ-1 U2OS cells after MMS (A) or CPT (B) treatment. Cells were treated with MMS (0.01%, 1h) or CPT (10 μM, 1h), and were allowed to recover for 8 h. Scale bar, 10 μm. Data are presented as the mean ± SEM, n > 150 cells. *, $P < 0.05$; **, $P < 0.01$ (two tailed t-test). (C, D) Representative immunofluorescence images and quantification of γ-H2AX foci in DJ-1 KO and WT U2OS cells after MMS (C) or CPT (D) treatment. Cells were treated with MMS (0.01%, 1h) or CPT (10 μM, 1h), and were allowed to recover for 8 h. Scale bar, 10 μm. Data are presented as the mean ± SEM, n > 150 cells. *, $P < 0.05$; **, $P < 0.01$ (two tailed t-test). (E, F) Representative images of tail moments in DJ-1 KO (E) or stable expression of DJ-1 (F) U2OS cells treated with ETO (10 μM, 1 h) as determined by a neutral comet assay. Scale bar, 10 μm.

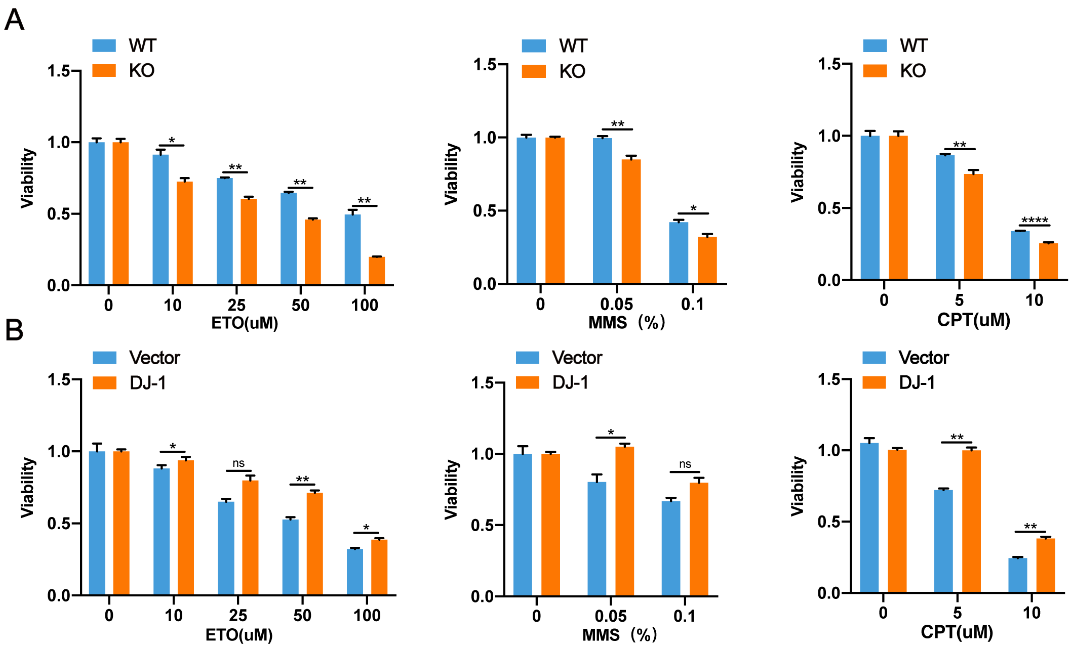

Figure S4. DJ-1 protects cells against genotoxic agents. (A) Viabilities of DJ-1 KO and WT U2OS cells after treatment with ETO (24h), MMS (6h) and CPT (12h) as indicated doses. Cell viability was determined by CCK-8 assays. Data are presented as the mean ± SEM, n = 5. *, $P < 0.05$; **, $P < 0.01$; ****, $P < 0.0001$ (two tailed t-test). (B) Viabilities of WT and stable expression of DJ-1 U2OS cells after treatment with ETO (24h), MMS (6h) and CPT (12h) as indicated doses. Cell viability was determined by CCK-8 assays. Data are presented as the mean ± SEM, n = 5. *, $P < 0.05$; **, $P < 0.01$; ns, nonsignificant (two tailed t-test).

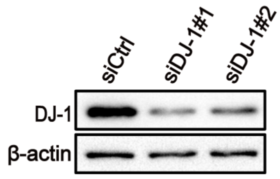

Figure S5. Verification of the siRNA of DJ-1 used in this study. Different siRNAs targeting human DJ-1 were transfected into 293T cells for 72 h and cell lysate was collected for western blot.

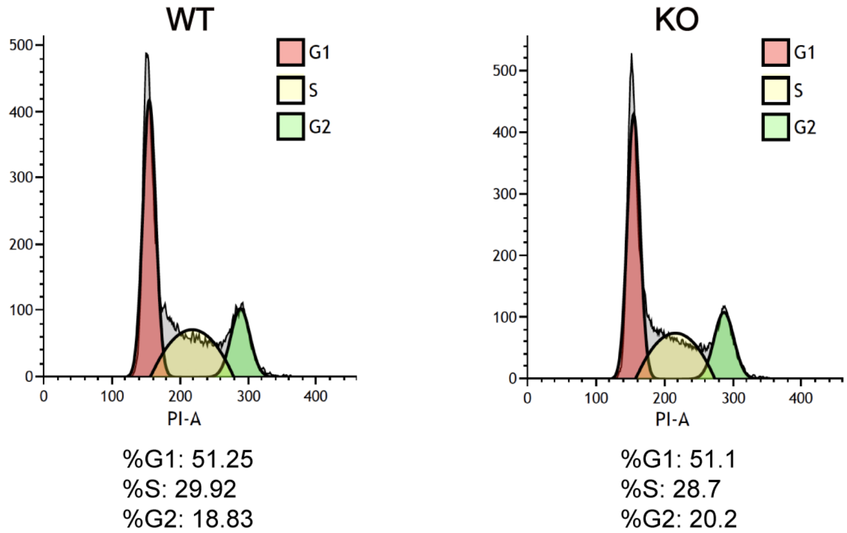

Figure S6. Depletion of DJ-1 in U2OS cells does not significantly change the cell cycle distribution. Representative cell cycle profiles of DJ-1 WT vs KO cells. Histogram data are representative of 3 independent experiments.

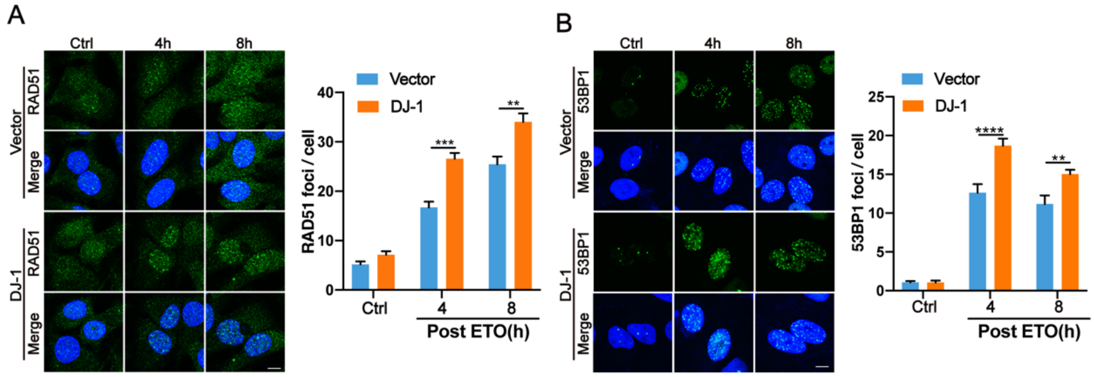

Figure S7. DJ-1 increases RAD51 and 53BP1 foci after DNA damage. (A) Representative immunofluorescence images and quantification of RAD51 foci in WT and stable expression of DJ-1 U2OS cells treated with ETO (10 µM, 1 h) at the indicated time points. Scale bar, 10 µm. Data are presented as the mean ± SEM, n > 150 cells. **, $P < 0.01$; ***, $P < 0.001$ (two tailed t-test). (B) Representative immunofluorescence images and quantification of 53BP1 foci in WT and stable expression of DJ-1 U2OS cells treated with ETO (10 µM, 1 h) at the indicated time points. Scale bar, 10 µm. Data are presented as the mean ± SEM, n > 150 cells. **, $P < 0.01$; ****, $P < 0.0001$ (two tailed t-test).

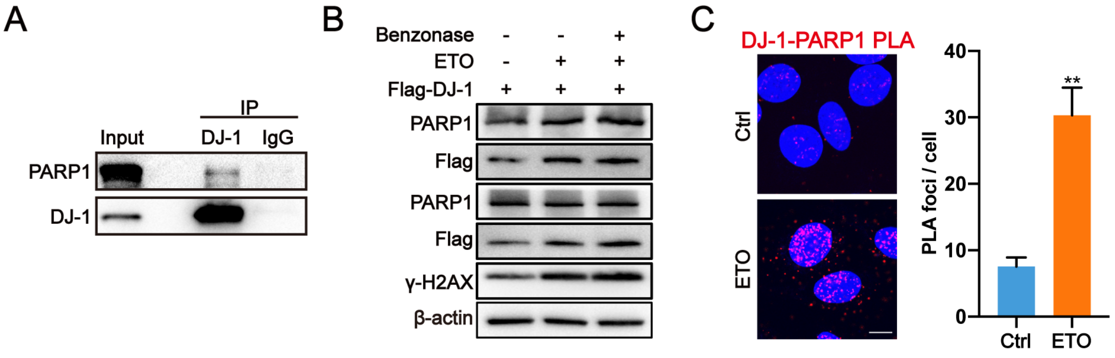

Figure S8. DJ-1 interacts with PARP1 in response to DNA damage**.** (A) Representative endogenous co-immunoprecipitation (Co-IP) analysis of DJ-1 and PARP1 in HEK293T cells. (B) Representative Co-IP analysis of DJ-1 and PARP1 after ETO (10 µM, 1h) in the presence of Benzonase in HEK293T cells. (C) Representative PLA signals between DJ-1 and PARP1 in U2OS cells following ETO (10 µM, 1 h) treatment. Quantification of average PLA signals was shown at right. Scale bar, 10µm. Data are presented as the mean ± SEM, n > 100 cells. **, *P* < 0.01 (two tailed t-test).

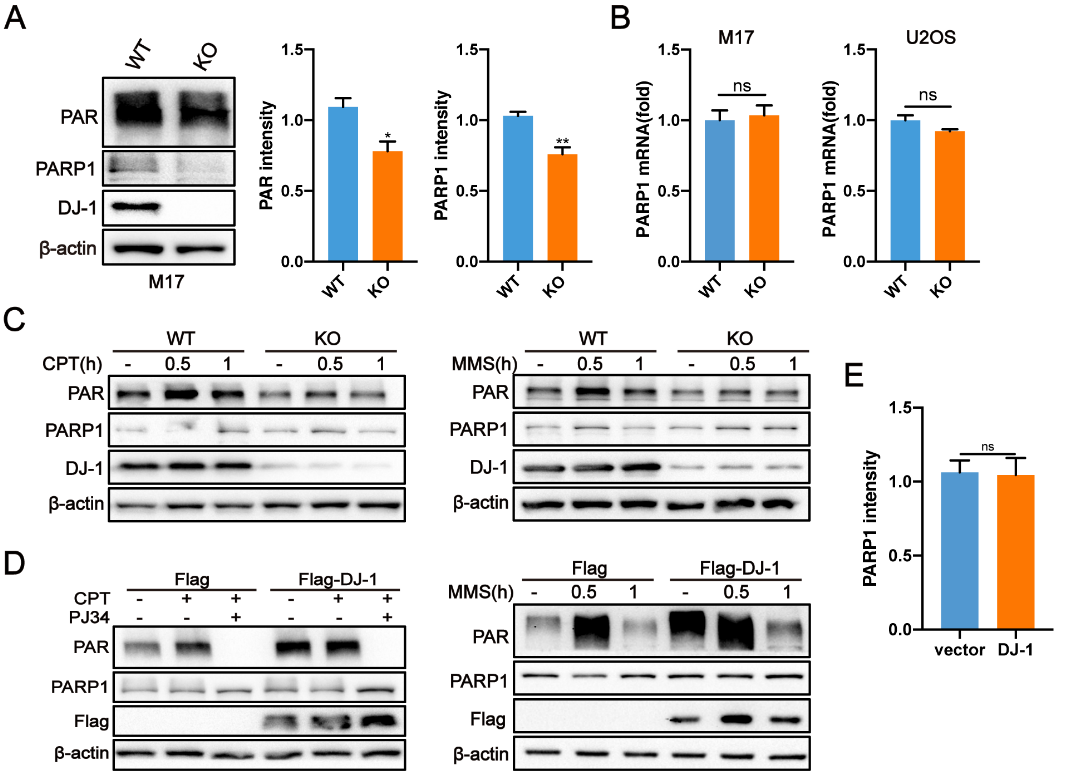

Figure S9. DJ-1 facilitates PARP1 activity upon DNA damage. (A) Representative western blot and quantification of PAR and PARP1 proteins in DJ-1 KO and WT M17 cells. Data are presented as the mean ± SD, n = 3. *, *P* < 0.05; **, *P* < 0.01 (two tailed

t-test). (B) Transcript levels of PARP1 were determined by qPCR in WT and DJ-1 KO M17 (left) and U2OS (right) cells. GAPDH was used to normalize expression. Data are presented as the mean ± SEM, n=3. ns, nonsignificant (two tailed t-test). (C) Representative western blot of PAR and PARP1 in DJ-1 KO U2OS cells treated with CPT (10 μM, left), or MMS (0.01%, right) at the indicated timepoints. n=3. (D) Representative western blot of PAR and PARP1 in U2OS expressing Flag-DJ-1 treated with CPT (10 μM, left) and MMS (0.01%, right) in the presence and absence of PJ34 (10 μM). n=3. (E) Quantification of PARP1 expression in U2OS cells expressing Flag-DJ-1. Data are presented as the mean ± SD, n = 3. ns, nonsignificant (two tailed t-test).

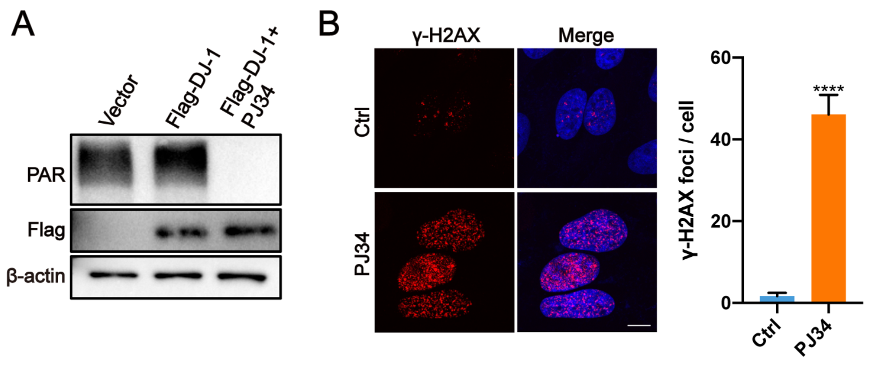

Figure S10. PARP1 inhibition by PJ34 results in DNA damage accumulation. (A) Representative western blot of PAR in HEK293T cells expressing Flag-DJ-1 treated with PJ34. (B) Representative immunofluorescence images and quantification of γ-H2AX foci in U2OS cells treated with PJ34 (10 μM, 12 h). Scale bar, 10 μm. Data are presented as the mean ± SEM, n > 150 cells. ****, $P < 0.0001$ (two tailed t-test).

Table S1. qPCR primers and siRNA sequences

Human qPCR primers	Forward and reverse oligo. sequences	siRNA sequences	
DJ-1-F	GTCCTACTGCTCTGTTGGCTCA	DJ-1 siRNA-1-sense	AGCAGACUCAGAUAAAUUCUGUGC
DJ-1-R	CCACACGATTCTCAGAGTAGGTG	DJ-1 siRNA-1-antisense	CGCACAGAAUUUAUCUGAGUCUGC
PARP1-F	CCAAGCCAGTTCAGGACCTCAT	DJ-1 siRNA-2-sense	CCAGCUUCGAGUUUGCGCUUGCAA
PARP1-R	GGATCTGCCTTTTGCTCAGCTTC	DJ-1 siRNA-2-antisense	AUUGCAAGCGCAAACUCGAAGCUG
GAPDH-F	GTCTCCTCTGACTTCAACAGCG		
GAPDH-R	ACCACCCTGTTGCTGTAGCCAA		
